# Mediterranean Diet Adherence, Gut Microbiota and Parkinson’s Disease: A Systematic Review

**DOI:** 10.3390/nu16142181

**Published:** 2024-07-09

**Authors:** Bibi Aliya Seelarbokus, Elisa Menozzi, Anthony H. V. Schapira, Anastasia Z. Kalea, Jane Macnaughtan

**Affiliations:** 1 Division of Medicine, University College London (UCL), London WC1E 6JF, UK; aliya.seelarbokus.22@alumni.ucl.ac.uk (B.A.S.); a.schapira@ucl.ac.uk (A.H.V.S.); 2 Department of Clinical and Movement Neurosciences, UCL Queen Square Institute of Neurology, London WC1N 3BG, UK; 3 Aligning Science Across Parkinson’s (ASAP) Collaborative Research Network, Chevy Chase, MD 20815, USA; 4 Institute for Liver and Digestive Health, University College London, Royal Free Campus, London WC1E 6JF, UK

**Keywords:** Parkinson’s disease, Mediterranean diet, microbiota, cognition, gastrointestinal symptoms

## Abstract

Background: There is mounting evidence to suggest that high adherence to the Mediterranean diet (MedDiet) may reduce the risk of age-related diseases, including Parkinson’s disease (PD). However, evidence for the role of the MedDiet in the relief of motor and non-motor symptoms in patients with PD remains limited and inconclusive. We provide a systematic review of the effects of the MedDiet on the clinical features of PD using data from randomised controlled trials (RCT) and prospective observational studies. Methods: We searched MEDLINE, EMCare, EMBASE, Scopus and PubMed from inception until June 2023. Reference lists and the grey literature were also searched. Human studies with no restriction on language or publication date, examining associations between MedDiet adherence and the symptoms of PD, were included. We employed standard methodological procedures for data extraction and evidence synthesis and used the Quality Criteria Checklist for assessing the studies included. Results: Four studies from three unique cohorts, including two observational studies (n = 1213) and one RCT (n = 70), met the inclusion criteria. Despite the short study duration reported in all included reports, high MedDiet adherence was associated with changes in the gut microbiota (e.g., increased abundance of short-chain fatty acids producers). These outcomes correlated with a significant improvement in several non-motor symptoms including cognitive dysfunction, dyspepsia and constipation. However, there were no significant changes in diarrhoea, gastrointestinal reflux, abdominal pain and motor symptoms. Conclusion: High MedDiet adherence may be associated with significant improvement in global cognition and several gastrointestinal symptoms, possibly associated to changes in gut microbiota composition. Further studies are warranted to clarify potential cause-and-effect relationships and to elucidate MedDiet impact on motor symptoms.

## 1. Introduction

Parkinson’s disease (PD) is the second most common neurodegenerative disease after Alzheimer’s disease (AD), with a global prevalence of 8.5 million patients with PD reported in 2019 [1]. In the United Kingdom, despite acknowledging the possibility of clinical diagnostic mis-classification with error rates ranging from 15% to 24% in neurodegenerative diseases [2], the prevalence and incidence rates of PD in 2020 have been estimated at 286.5 per 100,000 person years and 33.4 per 100,000 person years, respectively [3,4]. Alongside a ratio of healthy ageing life expectancy (HALE) to LE of 0.8 [5], ageing remains the primary risk factor for PD [6] and, in fact, accounts for one additional year of life per person being lost to disability every 15 years [7]. On the current trajectory, at a median age of PD development at 60 years, PD cases are expected to double by 2030 [8]. Considering the distress caused to patients and families, in addition to the social and health care cost of PD, estimated to exceed GBP 16, 582 per affected person in the UK [9], the implementation of personalised or public health strategies seeking to target modifiable risk factors represents an urgent public health and research priority.

**Figure 1:**
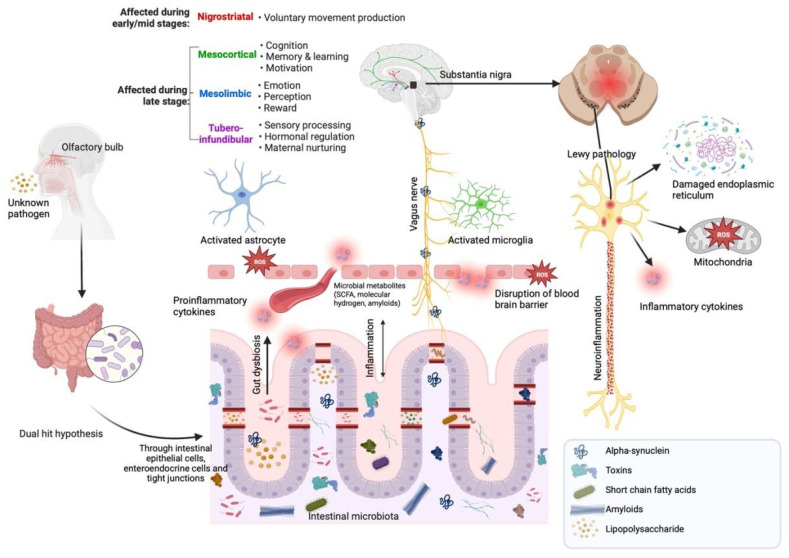
Overview of the gut–brain axis in the pathogenesis of Parkinson’s disease. Figure shows dysbiosis in the intestinal microbiota which leads to the translocation of bacterial metabolites, including alpha-synuclein, toxins, short chain fatty acids and lipopolysaccharides across the blood–brain barrier which separates the CNS and the blood vessel lumen. Misfolded α-synuclein which may be induced by the accumulation of inflammatory cytokines and ROS propagates in bottom-up fashion to the neurons in the brain via the vagus nerve. The accumulation of α-synuclein aggregates in the brain is also associated with Lewy pathologies, which in turn self-sustain inflammation and accumulation of ROS. Figure has been created using https://BioRender.com (accessed on 4 April 2024).

At its core, PD is a complex progressive neurodegenerative disease hallmarked by the early death of dopaminergic neurons in the substantia nigra pars compacta (SNpc) [10]. Once around 50% of substantia nigra dopamine neurons within the basal ganglia have been lost, classical parkinsonian motor symptoms including bradykinesia, 4–6 Hz rest tremor and muscular rigidity start to manifest while postural instability and accumulative gait disturbances drive the progress of motor disability [11,12]. However, considering that the pathogenic process also disturbs the peripheral nervous system in virtually all patients [13], a constellation of non-motor symptoms such as constipation, olfactory dysfunction, cognitive decline and depression and gastrointestinal (GI) impairment has also been associated with PD [10,14]. Interestingly, those findings have raised the possibility that the spread of Lewy bodies might not be exclusively bound to neuronal damage in upper brain regions associated with motor symptoms [15,16,17]. This idea of a gut–brain axis has subsequently been reinforced by the “dual-hit hypothesis”, postulating that sporadic PD begins when an unidentified neurotropic pathogen enters the body either through the nasal or the gastric route, before being retrogradely carried from the enteric nervous system (ENS) into the CNS, where trans-synaptic spreading across susceptible brain regions then occurs [18,19] (Figure 1).

PD is very likely heterogeneous in aetiology, although genetic causes are the only ones identified to date [20]. Nevertheless, environmental factors may play a role in modifying the risk for PD [20]. Treatment to date remains symptomatic and does not influence the progressive neurodegeneration associated with PD. Whereas dopaminergic drugs are the mainstay of symptomatic therapy, they have little influence on the non-motor features of PD which can often predate the onset of motor symptoms by up to 20 years [13]. Importantly, given that the gastrointestinal origin of the pathological protein aggregate, α-synuclein, in PD is thought to be the enteroendocrine cell involved in sensing end products of digestion, there is now an emerging interest in dietary manipulation as a therapeutic strategy in modulating the natural history of PD [21].

**Figure 2:**
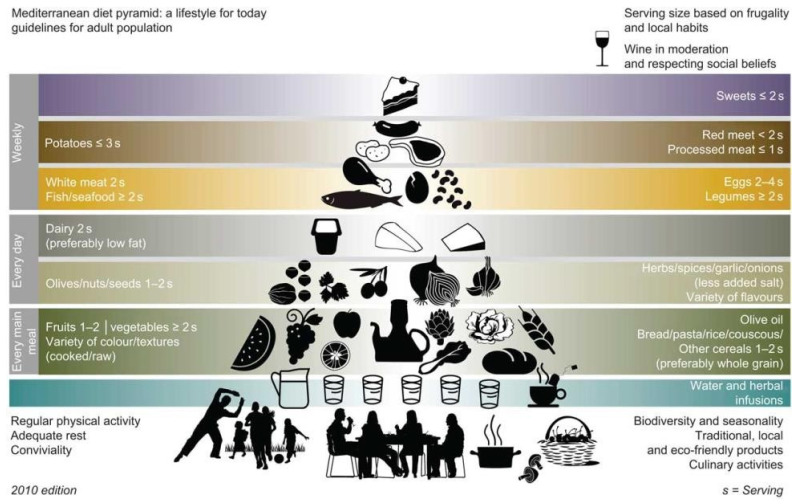
The diagram shows the modern Mediterranean diet pattern which emphasises a high intake of plant-based foods forming the base of the pyramid, and a low-intake of animal-based products and foods rich in salt, sugar and saturated fat. The pyramid also includes components of moderate physical activity, adequate rest, conviviality and biodiversity which are part of a Mediterranean lifestyle. Adapted from [38].

Worldwide, the Mediterranean diet (MedDiet), traditionally characterised by a high intake of seasonally fresh and locally grown plant-based foods, moderate consumption of fish and red wine, minimal consumption of red meat and processed foods along with the consistent use of cold-pressed olive oil as the principal cooking fat (Figure 2), has been heralded as one of the healthiest dietary patterns for its crucial role in the prevention and alleviation of chronic age-related pathological morbidities [22,23,24]. Specifically, owing to strong anti-oxidative and anti-inflammatory properties, the synergistic effects of polyphenols, polyunsaturated fatty acids (PUFAs), B vitamins, minerals, dietary fibre, and antioxidants found in whole grains, fruits, vegetables, nuts and red wine have been associated both with reduced symptomatic effects and risk of PD [25].

A protective effect of the MedDiet in neurodegeneration has been reported by several prospective studies. One of the more rigorous studies examining the effect of the MedDiet on health outcomes is the large Prevención con Dieta Mediterránea (PREDIMED) trial in older Spanish adults, whereby participants consuming a low-fat diet, enriched with nuts and olive oil had significantly increased levels of brain derived neurotropic factor (BDNF), lower concentrations of inflammatory biomarkers and better neurocognitive outcomes, compared with baseline values and with the placebo group [26,27]. In addition, a positive association between the MedDiet and risk of PD has been reported in the largest prospective study of dietary patterns analysing a male cohort from the Health Professionals Follow-Up Study (HPFS) and a female cohort from the Nurses’ Health Study (NHS) over a 16-year follow-up period in the United States at a pooled multivariate-adjusted relative risk (RR) for the top compared with the bottom quintiles of the calculated MedDiet score at 0.78 (95% CI: 0.56, 1.07; *p* for trend = 0.04) [28]. These findings corroborated with methodologically robust studies including the Rotterdam Study involving 9414 participants [29] and a cohort study of 41,715 Swedish women whereby each 1-point increase in the adherence score from a 10-unit scale devised by [30] was accompanied by a 29% reduced risk of PD in individuals aged 65 years and above [31].

Although there is a body of evidence of epidemiological prospective studies which reports the MedDiet as a protective mechanism against PD development [24,32,33], the benefits of the MedDiet in patients with manifest PD is less certain. For example, whereas Agarwal and colleagues [34] reported that a higher adherence to the MedDiet slowed PD progression, Maraki and colleagues found no significant change in symptoms among 34 cases with PD participating in the Hellenic Longitudinal Investigation of Aging and Diet, although noting that their analysis might have lacked statistical power to detect significance [35].

To the best of our knowledge, no study to date has comprehensively reviewed the effect of the MedDiet on motor and non-motor symptoms of patients with PD. Moreover, diet has been recognised as an important modulator of the gut microbiota composition and a key regulator of endotoxins, inflammatory cytokines and reactive oxygen species (ROS), and there is accumulating evidence favouring the existence of a significant alteration in the gut microbiota composition in patients with PD [36,37]. The aim of this review is to critically assess the effectiveness of the MedDiet on the symptomatic effects in patients with PD.

## 2. Methods

### 2.1. Search Strategy

A systematic-type literature search was conducted to identify publications from inception to June 2023 under the topic of this review. Five databases, Medline, EMBASE, PubMed, EMCare and Scopus, were searched. The searching strategy was first developed in Medline using medical subject heading (MeSH) terms or equivalent, text word terms and related keywords, and the whole searching process was under the supervision of an expert librarian. We used adaptations of it for EMBASE, EMCare, PubMed and Scopus to capture studies evaluating the effects of MedDiet adherence on PD symptoms and/or progression. Reference lists of selected studies, relevant websites, journals and the grey literature using Index to Theses were also searched to identify any additional relevant studies. To check for ongoing trials, we also searched ClinicalTrials.gov (www.clinicaltrials.gov). Detailed searching strategy and terminology can be found in the Supplementary Materials.

### 2.2. Inclusion and Exclusion Criteria

The link between the MedDiet and PD has not been extensively studied in the literature through RCTs, hence additional levels of evidence were also considered in this review, with no restrictions in place regarding the study duration, sample size, language, participants’ age or sex to increase sensitivity. Therefore, based on our study inclusion criteria, prospective observational studies were considered in addition to any RCTs, but all study designs were reported separately.

Adults of all ages (18 years and above) who met the diagnostic criteria for PD according to the International Classification of Diseases for Parkinson’s disease were considered [39]. Eligible studies included designs which applied a MedDiet intervention and which prospectively tested the intervention to a group of participants. Although different variants of the MedDiet across different countries were accepted, at least three of the following components were essential to meet the definition of a MedDiet-style diet [40], including a high ratio of monounsaturated to saturated fat, a high intake of fruits, vegetables, whole grains, cereals and legumes, a low to moderate consumption of poultry and dairy, an increased consumption of fish, a low intake of red meat and a low to moderate consumption of red wine. The control group, we defined as either no intervention or by adherence to the habitual dietary pattern specific to the country of interest.

Our exclusion criteria were retrospective observational studies, studies on experimental animal models of PD and studies including patients both with PD and severe mental illness because neurocognitive deficits are present in mental diseases and may represent an important confounding factor. To avoid the possibility of confounding and potential biases, studies which included multi-domain interventions such as physical activity, social support, and/or other dietary interventions, among others, were excluded, unless the effects of the MedDiet intervention were analysed separately.

### 2.3. Data Extraction

Two reviewers (B.A.S. and J.M.) screened the articles at different time points, and consensus was reached for excluded studies after discussion with a third reviewer (A.Z.K.). Firstly, studies were excluded based on title and abstract; then, full-text screening was conducted following the inclusion/exclusion criteria. Finally, critical characteristics of selected papers were extracted using the following categories: (1) country, (2) study design, (3) participant characteristics (number of subjects randomised, age, gender, diagnostic criteria for PD, time since PD diagnosis), (4) intervention and control characteristics, (5) main findings, (6) study quality, and (7) additional notes and funding sources.

### 2.4. Data Collection and Analysis

Search results were exported into EndNote software (version X20, Thomson Reuters, Philadelphia, PA, USA) and duplicates were manually removed. Titles and abstracts were screened using the online service of Rayyan [41]. Subsequently, a more comprehensive full-text screening was conducted according to the a priori selection criteria of eligible studies for this review. In case of disagreements, an additional reviewer (A.Z.K. or J.M.) arbitrated.

### 2.5. Assessment of Risk of Bias in Included Studies

To avoid bias and have a deeper understanding of the limitations of the selected studies, the quality of eligible studies was critically assessed using the Quality Criteria Checklist created by the Academy of Nutrition and Dietetics for primary research by B.A.S. [42]. These checklists were derived and included items addressing issues related to the research question, population, intervention, outcomes, confounding factors, follow-up and statistical analyses. A risk of bias table for each study was created, and the studies were assessed as positive, neutral or negative (see Supplementary Materials). Any discrepancies were resolved by discussion with an additional reviewer (A.Z.K. or J.M.) where necessary.

### 2.6. Assessment for Heterogeneity and Synthesis of the Evidence

We intended to use the *I*^2^ statistic to estimate statistical heterogeneity between studies. A meta-analysis was not possible due to the varied study designs considered coupled with the small number of studies per study design. Hence, only methodological heterogeneity was assessed at all steps of this review and reported as a narrative of the synthesis of the evidence, while taking the opportunity to highlight any gaps in the literature.

## 3. Results

### 3.1. Results of the Search

**Figure 3:**
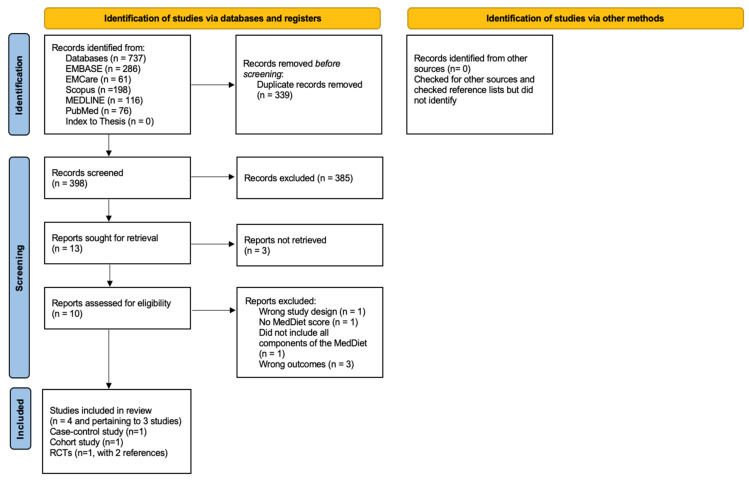
Preferred Reporting Items for Systematic Reviews and Meta-Analyses (PRISMA) flowchart of study selection process.

The initial comprehensive search (24 April 2023) identified a total of 737 records, of which 339 duplicates were excluded. With no additional papers found in the Grey Literature Report database and in the updated search (June 2023), 398 titles and abstracts were screened, and 13 papers were eligible for full-text screening but only 10 papers were accessible for complete text review. The detailed Preferred Reporting Items for Systematic Reviews and Meta-Analyses (PRISMA) flowchart, with reasons of excluding studies, is shown in Figure 3.

### 3.2. Excluded Studies

Out of ten full-text studies assessed for eligibility, six studies were excluded. Three studies did not measure the effect of MedDiet components on PD correctly, and the remaining three studies were either of a retrospective observational study design or did not meet the criteria or score defining a typical high MedDiet adherence. Details and reasons for the exclusion for the specific studies which missed the inclusion criteria are presented in the Supplementary Materials.

### 3.3. Included Studies

A total of four references representing three unique cohorts, all published from 2020 onwards, were included in this systematic review. The outcomes were explored using evidence from RCTs and observational studies, and each level of evidence is presented separately.

One cohort study and one case-control study from the United States (US), in addition to one single-blind RCT from Iran with two references, were retrieved. All studies included both sexes, with the proportion of males being slightly higher. Overall sample sizes ranged from 8 to 1205 subjects, with no restriction placed on the ethnic group, education or income levels of the study participants. The studies by Paknahad and colleagues [43,44] and Rusch and colleagues [45] compared the outcomes between the MedDiet intervention and control group over five to ten weeks, while Fox and colleagues [46] only reported the effects of the MedDiet in patients with PD, with no indication regarding the duration of the adherence period.

### 3.4. Characteristics of the Included Studies

#### 3.4.1. Randomised Controlled Trials (RCT)

Table 1 describes the design aspects of the RCT, with each reference reporting different outcomes of interest after ten weeks of MedDiet intervention. MedDiet adherence was defined by a list of foods constituting the traditional MedDiet pyramid (dairy, meat, fruits, vegetables, legumes, cereals and fish) which was personalised based on the anthropometric characteristics, age and calorie requirements of each study participant. Within the control group, the study population adhered to an Iranian diet, traditionally consisting of 12% protein, 58% carbohydrate and 30% fat of the total calories [43]. However, given that all study participants were from a single Iranian centre, the findings cannot be generalised to other areas of Iran and the world.

With 10–12.5% of participants lost to follow-up in each arm, the sample size ranged between 34 and 36 subjects per randomisation arm. In the intervention group, study participants, of whom 55.6% were males, had a mean ± SD age of 59.3 ± 8.3 years, body mass index (BMI) at 25.8 ± 3.3 kg/m^2^ and a disease duration of 6.6 ± 6.02 y. Considering a very similar spread of the study population in the control group where 61.8% of participants were males, had a mean ± SD age of 58.6 ± 9.3 years, BMI of 25.3 ± 2.7 kg/m^2^ and a disease duration of 5.8 ± 4.9 y, there were no significant differences between groups (*p* > 0.05).

#### 3.4.2. Case-Control Study

Table 2 provides an overview of the characteristics of the case-control study conducted in eight patients with PD who adhered to a 2-week baseline control diet followed by a MedDiet intervention diet for five weeks. In this study, MedDiet adherence was assessed using the 14-item Mediterranean Diet Adherence Screener (MEDAS) questionnaire, and the association between higher vs. lower MedDiet adherence was studied based on the changes in gut microbial communities and GI symptoms in PD. Briefly, the MEDAS is a well-validated 14-item scoring tool for non-Mediterranean countries where a score ≥ 10 indicates “good adherence” [47].

The study population was exclusively Caucasians, with more males (63%) and had a mean ± SD age of 71.4 ± 2.6 yr. At a mean ± SD BMI of 26.7 ± 1.4 kg/m^2^, most of the study participants were considered overweight according to the World Health Organization’s criteria for Caucasian populations [48]. With no restrictions in place regarding the use of medications throughout the study period, all the participants were taking levodopa but only three patients reported the concurrent use of monoamine oxidase-B inhibitors.

#### 3.4.3. Cohort Study

As part of the Modifiable Variables in Parkinsonism (MVP) Study, Fox and colleagues [46] analysed the effects of the MedDiet (measured using the 14-item MEDAS questionnaire) on PD severity using the PRO-PD score, as described by Mischley and colleagues [49].

As shown in Table 3, from 1205 included participants, the study population was predominantly Caucasian (91%), female (59%) and the mean ± SD age was 66.4 ± 8.76 yr. At an average MEDAS score of 7.8, the total PRO-PD score improved by 25.6 (37.2–14.0) points for each 1-unit point increase in the MedDiet score (R^2^ = 0.1982, *p* < 0.001) after adjustment for age, gender, income and years since diagnosis. The non-motor PRO-PD sub-score decreased by 13.0 (19.1–6.94) points for each 1-unit increase in the MedDiet score (R^2^ = 0.1582, *p* < 0.001) whereas the motor PRO-PD sub-score decreased by 9.78 (14.3–5.23) points per point increase in MedDiet score (R^2^ = 0.2173, *p* < 0.001).

### 3.5. Results of the Quality Assessment

As presented in Table 4, all of the included studies received a positive quality rating, suggesting a low risk of bias, and the internal validity of each study was robust for MedDiet adherence in patients with PD. However, all studies except the study by Fox and colleagues [46] were small.

### 3.6. Outcomes

#### 3.6.1. Evidence from Randomised Controlled Trials (RCTs)

##### Effect of the Mediterranean Diet (MedDiet) on Global Cognitive Function and Specific Cognitive Domains

In the study by Paknahad and colleagues, a significant decrease in energy, carbohydrate, saturated fat and total fat intake was noted after a 10-week high adherence to the MedDiet [43]. Although not significant, the intake of polyunsaturated fatty acid paradoxically decreased in the MedDiet intervention group. In contrast, the intake of protein, eicosapentaenoic acid and linoleic acid was significantly increased in the intervention group. From the Montreal Cognitive Assessment (MoCA) test score, those changes were associated with significant improvements in executive function (intervention group: 0.74 ± 0.21 vs. control: −0.03 ± 0.06, *p* = 0.001, n = 35), language score (intervention group: 0.53 ± 0.26 vs. control: −0.05 ± 0.04, *p* = 0.02, n = 35), concentration and working memory (intervention group: 0.32 ± 0.13 vs. control: −0.08 ± 0.85, *p* = 0.04, n = 35), and the global cognitive assessment score (intervention group: 1.61 ± 2.25 vs. control: −0.61 ± 1.11, *p* = 0.001, n = 35). However, there was no significant change observed in visuospatial abilities (*p* = 0.99), short-term memory recall (*p* = 0.30) and orientation to time and place (*p* = 0.24) from the general linear model univariate analysis.

##### Effect of the Mediterranean Diet (MedDiet) on Serum Total Antioxidant Capacity, Motor and Non-Motor Symptoms beyond Cognitive Dysfunction

Paknahad and colleagues compared the effects of the MedDiet vs. the traditional Iranian diet on the serum total antioxidant capacity (TAC) from the cumulative contribution of vitamin E, vitamin C, selenium and β-carotene [44]. After ten weeks of high adherence to the MedDiet, a significant increase in the intakes of selenium (*p* = 0.04) and β-carotene (*p* = 0.002) was reported, but there were no significant changes in the intakes of vitamin E (*p* = 0.68) and vitamin C (*p* = 0.32) between the MedDiet group and the control group. However, with a significant increase observed in total serum TAC concentrations (*p* < 0.001), there were also improvements in mentation, behaviour and mood (*p* = 0.03); activity of daily living (i.e., speech, salivation, ingestion, hand-writing, walking, dressing and personal hygiene) (*p* = 0.003); and complications of therapy (*p* = 0.04), as assessed by the MDS-UPDRS score constituting four parts, namely, I: Non-motor Experiences of Daily Living; II: Motor Experiences of Daily Living; III: Motor Examination; and IV: Motor Complications [50]. Whilst these findings suggest that the MedDiet could contribute to the relief of non-motor symptoms of PD, there was no significant relation between MedDiet adherence and motor examination (e.g., resting tremor, bradykinesia, rising from the chair, stability and standing) (*p* = 0.80).

#### 3.6.2. Evidence from the Case-Control Study

##### Mediterranean Diet (MedDiet) Adherence, Microbial Communities and Gastrointestinal Function

At a mean ± MEDAS score of 4.4 ± 0.6 in the MedDiet intervention group, there was a significant increase in carbohydrate density (% total calories (kcal)), dietary fibre, total fat, total fat density, monounsaturated fat (including oleic acid) and polyunsaturated fat (including linoleic acid), while cholesterol intake was significantly reduced. These changes corresponded to a significant reduction in body weight by 2.5 kg. After five weeks of high adherence to the MedDiet, the abundance of Proteobacteria was significantly increased (5.8 ± 1.6%; *p* = 0.01). Whilst the proportion of *Roseburia* was significantly lower in patients with PD compared to controls prior to the intervention (0.6% ± 0.2 vs. 1.6% ± 0.3, *p* = 0.03), a significant increase was reported at week 5 (0.9 ± 0.2%, *p* < 0.01). On the other hand, the proportion of *Desulovibrionaceae* was significantly higher at baseline in patients with PD compared to controls (1.1 ± 0.2% vs. 0.3 ± 0.1%; *p* < 0.01) and decreased after five weeks of the intervention (0.9 ± 0.2%, *p* = 0.04). In addition, the prevalence of *Clostridium bolteae*, *Ruminococous*, *Blautia*, *Dorea* and *Lachnospiraceae* decreased after the diet intervention, suggesting the role of the MedDiet in the modulation of the gut microbiota in PD. Those changes corresponded with significantly reduced symptoms of constipation (baseline: 2.25 ± 0.48 vs. intervention: 1.54 ± 0.31, *p* = 0.04) and dyspepsia (baseline: 1.69 ± 0.21 vs. intervention: 1.41 ± 0.15, *p* = 0.02), as measured by the Gastrointestinal Symptom Rating Scale (GSRS) ranging from a score of 1 to 7, where 1 represents no discomfort at all and 7 represents very severe discomfort.

#### 3.6.3. Evidence from the Cohort Study

##### Non-Motor Symptoms in Parkinson’s Disease (PD)

When analysed per specific symptom experienced by patients with PD, higher MedDiet scores were significantly associated with a decrease in non-motor symptoms of constipation (*p* = 0.0005), motivation (*p* < 0.001), depression (*p* = 0.003), withdrawal (*p* < 0.001), anxiety (*p* = 0.01), fatigue (*p* = 0.04), daytime sleepiness (*p* = 0.001), visual disturbances (*p* = 0.03), insomnia (*p* = 0.002), muscle pain (*p* = 0.003), forgetfulness/memory (*p* < 0.001), comprehension (*p* = 0.002) and sexual dysfunction (*p* = 0.02), after adjustments for age, sex, income and years since diagnosis. However, given that the PRO-PD tool requires computer access and good literacy, these results might not be representative of the wider population of patients with PD [49].

## 4. Discussion

### 4.1. Principal Findings

This systematic review investigated the association between MedDiet adherence and the symptomatic effects of PD from the analysis of one small RCT with two references, one case-control study and one cohort study. Whilst a number of meta-analyses and systematic reviews investigating the effects of MedDiet adherence on the risk of PD have previously been published [51,52,53,54,55], this review differed in terms of the targeted population, whereby the effects of the MedDiet were studied in patients with manifest PD. To our knowledge, this is the first systematic review to comprehensively evaluate the effects of the MedDiet both on motor and non-motor symptoms of PD. Despite the current study being restricted to a qualitative analysis of only four references representing three unique cohorts, our findings demonstrated that a short-term adherence to the MedDiet of either five weeks or ten weeks could mediate changes in cognitive and GI functions, including significant positive associations observed in executive function, working memory, language, global cognitive function, constipation and dyspepsia in patients with PD. However, in line with a recent meta-analysis and systematic review of randomised and cross-over studies investigating the effect of nutrition in PD [56,57], there were no significant associations between high MedDiet adherence and changes in motor symptoms, diarrhoea, abdominal pain, reflux syndrome, short-term memory and visuospatial abilities observed in this systematic review.

### 4.2. Effect of the Mediterranean Diet (MedDiet) on Motor Symptoms

In this study, the cumulative effect of the antioxidants of interest, i.e., selenium, vitamin C, vitamin E and β-carotene, was not associated with a significant improvement in the symptomatic effects in patients with PD. Considering that motor signs such as bradykinesia, rest tremor and rigidity typically manifest following the depletion of at least 50% dopaminergic neurons, one explanation underlying the non-significant association between higher levels of antioxidant and relief in motor symptoms could be the irreversible loss of dopaminergic neurons in manifest patients [11].

It is also plausible that the changes in the gut microbiota induced by the MedDiet may not influence levodopa bioavailability and absorption [58]. Given that in PD, the prevalence of constipation is elevated and associated with small intestinal overgrowth (SIBO) in 56% of patients using proton pump inhibitors [59], a high abundance of gut bacteria, mainly enterococci caused by SIBO, could negatively affect levodopa pharmacokinetics and result in no significant changes in motor signs, irrespective of a high MedDiet adherence [58]. On the other hand, when patients with PD were treated with rifaximin to eradicate SIBO, improvements in motor fluctuations were noted as measured by clinical scales or amount of “ON/OFF” times [60].

Alternatively, given that levodopa has been shown to cross the blood–brain barrier via the sodium-dependent antiporter, LAT1-4F2hc (SLC7A5-SLC3A2) expressed on endothelial cells [61], it is also possible that the amino acids acquired from the MedDiet such as phenylalanine, tryptophan and leucine may drive further competition for levodopa absorption when administered with meals and thus contribute to non-significant changes in motor symptoms [62,63].

### 4.3. The Link between Short-Chain Fatty Acids (SCFAs), Mediterranean Diet (MedDiet) Adherence and Gastrointestinal Disturbances in Parkinson’s Disease (PD)

Constipation is amongst the most common GI symptoms experienced by 66–79% of all patients with PD [64,65]. In this review, the significant relief in constipation observed in patients following the MedDiet could be attributed to the high intake of whole grains, legumes and dried fruits providing at least 14 g of fibre for every 1000 kcal per day [66]. This could be explained by the properties of indigestible fibres which have a high affinity for water to soften the stools and accelerate colonic transit through mechanical peristaltic actions caused by an increased stool volume and the production of gas (CO_2_, CH_4_, H_2_) [67,68].

Moreover, whilst patients with PD often show reduced abundance of SCFAs in stool, the MedDiet has been purported to increase SCFAs in patients with PD to a similar level as controls from the effective fermentation of dietary fibre [37,69]. Those findings could be reinforced by Rusch and colleagues whereby patients adhering the MedDiet had an increased population of *Desulovibrionaceae* and *Roseburia*, both of which are known for their fibrolytic activity and capacity to produce SCFAs, in particular acetate, propionate and butyrate, by the anaerobic fermentation of non-absorbed carbohydrate and, to a lower degree, of protein by colonic microbiota [45,70]. Mechanistically, in addition to a higher migration of mucosal cells alongside an improved proliferation and differentiation of healthy colonocytes [71], several studies have demonstrated an enhanced colonic barrier function after SCFA supplementation [72,73,74]. Thus, whilst indigestion may be characterised by damage to the intestinal lining caused by the excessive secretion of acids and triggers of inflammation, a healthier intestinal barrier may not only enhance mucosal healing but also reduce the possibility of colonisation and inflammation triggers by gut bacteria [71].

Besides the physiological functions detailed above, SCFAs may also exhibit anti-inflammatory effects in intestinal mucosa through the activation of G-protein coupled receptors and the inhibition of histone deacetylases (HDACs) in colonocytes and mucosal immune cells [75]. In intestinal epithelial cell (IEC) models, the activation of GPR109a by butyrate has been reported to suppress the abundance of adhesion molecules in inflammatory cells and endothelial cells and prevent chemotaxis of monocytes to the area of inflammation [76,77]. In addition, the activation of GPR43 by acetate and propionate has been shown to stimulate potassium efflux and hyperpolarisation in colonic epithelial cells, thereby activating the NLRP3 inflammasome which regulates the secretion of pro-inflammatory cytokines [78]. Furthermore, butyrate has recently been recognised to inhibit colonic inflammation in two predominant ways: (i) by reducing inflammation mediated by interferon-γ (IFN-γ) and (ii) by stimulating T-cell apoptosis and thereby eliminating the source of inflammation [79]. Similarly, the activation of GPR43 by acetate and propionate has been shown to stimulate potassium efflux and hyperpolarisation in colonic epithelial cells, thereby activating the NLRP3 inflammasome, which regulates the secretion of pro-inflammatory cytokines [78].

Due to their small size, SCFAs may also enter the cytoplasm or even the nucleus of eukaryotic cells by passive diffusion or active transport where they could elicit a HDAC inhibitory activity [80]. Considering that the inhibition of HDACs suppresses the nuclear transcription factor NF-κB in the mucosal immune system and promotes the differentiation of mucosal Treg cells, these mechanisms provide an alternative pathway whereby the release of anti-inflammatory IL-10 from an increased expression of Foxp3 from Treg cells could regulate inflammation [81,82,83].

These mechanisms provide short-term plausible pathways underpinning the significant change observed in gastrointestinal symptoms in addition to other non-motor symptoms following an increased concentration of SCFAs from MedDiet adherence. In effect, an ongoing small exploratory open-label pilot study is currently underway to assess SCFA-prodrug tributyrin, which has been identified as a potential therapy (ClinicalTrials.gov ID: NCT05446168).

### 4.4. The Effect of Mediterranean Diet (MedDiet) on Cognitive Function in Parkinson’s Disease (PD)

Although studies conducted in Mediterranean populations are not always comparable to non-Mediterranean populations owing to the considerable differences in dietary composition and lifestyle behaviours between countries [84], a positive association between high MedDiet adherence and global cognitive function was observed in this systematic review, which included study participants from Iran [43] and the USA [46]. Potential mechanisms for the role of the MedDiet in neurodegeneration has extensively been analysed, though primarily from observational studies [85]. Substantial evidence from epidemiological studies has highlighted the strong potential of omega-3 fatty acid docosahexaenoic acid (DHA) to inhibit the expression of cyclooxygenase and reduce the secretion of inflammatory prostaglandins (PGs), especially PGE2 [86]. Importantly, this anti-inflammatory effect has been reinforced by the mechanisms of neuro-protectin D1 (NPD1), a metabolic derivative of DHA that protects the brain against injury-induced oxidative stress through (i) the inactivation of caspase activation signalling pathways, (ii) inhibition of hyperphosphorylation of tau and (iii) regulation of the phosphoinositide 3-kinase (PI3K)/Akt cascade [87,88,89]. More recently, epidemiological evidence has also supported the neuroprotective role of eicosapentaenoic acid (EPA), the precursor to DHA, in PD. Of note, the higher levels of EPA have been reported to attenuate the concentration of 1-methyl-4-phenylpyridinium (MPP+) neurotoxin that acts by disturbing oxidative phosphorylation in mitochondria, suppressing complex I and reducing dopamine levels in the brain [90,91]. In addition, whilst diets rich in saturated fat may exacerbate endotoxemia and inflammation by either elevating the concentration of LPS, or by directly stimulating TLR4 receptors, the higher levels of dietary fibre, polyphenols and antioxidants obtained from the MedDiet have been acknowledged to reduce the concentration of Gram-negative bacteria and enhance gut barrier function [92,93]. Those positive changes have consequently been associated with a reduced translocation of inflammatory cytokines and lower oxidative stress, both of which could attenuate the progression of neurodegeneration in PD [94,95,96,97]. However, given that the duration of the intervention was only of ten weeks, longer-term studies are warranted to confirm the potential complex mechanistic pathways which could be involved. We also need to note here that when exploring the health benefits of the Mediterranean diet as a dietary pattern, one cannot assume that the benefits if its individual components have an additive effect, as often the synergistic effect of the individual components is greater than the additive effect.

### 4.5. Strengths and Limitations of this Systematic Review

This review included a robust and sensitive systematic search from five large databases which captured eligible human studies with no language or date limitations according to a well-defined criteria-based selection. In addition, we provided quality assessments and critical discussion of the studies retrieved using a comprehensive reference list. However, our systematic review had several important limitations.

First, the studies included in this review had relatively small sample sizes and this could have limited the statistical power to detect a true significant result. Second, the duration of the included studies was brief, with interventions lasting only five to ten weeks, which may not be sufficient to assess the long-term effects of the MedDiet. Third, even if positive associations between MedDiet adherence and global cognitive function were observed in this review, it is important to note that a high adherence to the MedDiet was strictly monitored in all four included studies of this systematic review as per their study protocols. This is a potentially useful consideration given that the large-scale Chicago Health and Aging Project (CHAP) [98] and other observational studies [99,100] have recently demonstrated that a concurrent intake of foods high in fat and sugar from a traditional westernised dietary pattern could either attenuate or outweigh the effects of the MedDiet. In addition, even if other large-scale studies such as the “European Prospective Investigation into Cancer and Nutrition” (EPIC-Norfolk) study reported significant cognitive and other health benefits of the MedDiet in neurodegenerative diseases for British adults [101], the estimates for the positive health outcomes appeared modest when compared to studies conducted in Mediterranean populations [102]. Therefore, considering that our systematic review was limited to only four studies conducted in Iran and the US, the findings should be cautiously interpreted in the absence of studies in more diverse populations, encompassing different geographic regions and ethnic backgrounds. Finally, although not specific to the included RCT and the observational studies in this systematic review, the possibilities of reverse causality and residual confounding cannot be excluded. Indeed, considering that a diagnosis of PD may induce changes in the dietary habits of patients, the planned comparison between the findings of this review and previous studies might not be accurate if a higher intake of fruit and vegetables has been upheld in diagnosed patients with PD within the control groups [103]. Consequently, this could lead to an erroneous interpretation of the result of the assigned intervention, rendering any associations between the MedDiet and PD invalid or biased towards the null. Nevertheless, the application of G-estimation to RCTs which considers both “assigned and received treatment simultaneously in a structural nested model” could be an effective solution to this dilemma [104].

### 4.6. Conclusions and Future Directions

To conclude, the present systematic review has supported the beneficial associations between the MedDiet and changes in non-motor symptoms, including global cognition, constipation and dyspepsia in PD. Based on the study findings, but also the limitations and heterogeneity in study design, we can make some recommendations for research priorities. Clearly, the lack of long-term studies investigating the effects of the MedDiet on cognitive function could compromise the precise evidence regarding the associations between the MedDiet and PD. To this end, long-term and methodologically robust RCTs and observational studies conducted both within and outside of the Mediterranean basin and evaluating the effects of the MedDiet on the symptoms of PD are warranted for future systematic reviews and meta-analyses. Notably, these studies should be substantiated by Mendelian randomisation studies aiming to establish potential “cause and effect” relationships, given that genetic risk plays an important role in the onset and progression of PD. However, considering the long prodrome of PD, equally important would be to determine a critical age window where the MedDiet could be the most prophylactic in patients in earlier and reversible stages of PD.

In addition, given that age, female gender and lifestyle factors are well-established risk factors for several GI symptoms and cognition, it would be important to account for key confounders in future studies assessing associations between the MedDiet and symptoms of PD to improve the methodological quality, validity, comparability and homogeneity of the results.

## Figures and Tables

**Table 1:** Study characteristics and summary of findings of the included RCT.

Study (Country)	Study Design	Type of Intervention	Type of Control	Sample Size of Intervention Group	Sample Size of Control Group	Severity Rating Scale Used	Mean Age, y	Males, %	PD Duration, y	Duration of Intervention	Adjustment	Main Findings—MedDiet Associated with:	Additional Notes	Quality Assessment Rating
Paknahad et al., 2020 [43] (Iran)	RCT	MedDiet	Typical Iranian diet	35	35	The Hoehn and Yahr scale and the Unified Parkinson Disease Rating Scale (UPDRS)	MedDiet: 59.3 ± 8.3 Control: 58.6 ± 9.3	MedDiet: 61.8 Control: 55.6	MedDiet: 6.6 ± 6.0; Control: 5.8 ± 4.9	10 weeks	Univariate analyses only	Executive function (*p* = 0.001); Language (*p* = 0.02); Attention, concentration, working memory (*p* = 0.04); global cognitive function (*p* = 0.001) No significant differences in: visuospatial abilities (*p* = 0.99), short term memory recall (*p* = 0.3), orientation to time and place (*p* = 0.24)	Cognitive function was assessed using the Montreal Cognitive Assessment (MoCA) test Funding status not mentioned	Positive
Paknahad et al., 2022 [44] (Iran)	RCT	MedDiet	Typical Iranian diet	36	34	The Hoehn and Yahr scale and the Unified Parkinson Disease Rating Scale (UPDRS)	MedDiet: 59.3 ± 8.3 Control: 58.6 ± 9.3	MedDiet: 61.8 Control: 55.6	MedDiet: 6.6 ± 6.0; Control: 5.8 ± 4.9	10 weeks	Univariate analyses only	Increased intakes of selenium (*p* = 0.04) and beta-carotene (*p* = 0.002), serum total antioxdiant capacity (*p* < 0.001), mentation, behaviour and mood (*p* = 0.03), activity of daily living (*p* = 0.003), complications of therapy (*p* = 0.04) and total UPDRS (*p* = 0.01) No significant changes for intakes in vitamin E (*p* = 0.68) and vitamin C (*p* = 0.32) and in motor symptoms (*p* = 0.8)	Funding status not mentioned	Positive

**Table 2:** Study characteristics and summary of findings of the included case-control study.

Study (Country)	Study Design	Type of Intervention	Type of Control	Sample Size of Intervention Group	Sample Size of Control Group	Severity Rating Scale Used	Mean Age, y	Males, %	PD Duration, y	Duration of Intervention	Adjustment	Main Findings—MedDiet Associated with:	Additional Notes	Quality Assessment Rating
Rusch et al., 2021 [45] (USA)	Case control	MedDiet	Baseline diet/usual diet	8	8	The Hoehn and Yahr scale and the Unified Parkinson Disease Rating Scale (UPDRS)	71.4 ± 2.6	63.8	Not specified	5 weeks	Univariate analyses only	Increase in Proteobacteria proportion; Decrease in Desulfovibrionaceae, Clostridium bolteae, Ruminococous, Blautia, Dorea, Lachnospiraceae (*p* < 0.01), improvement in constipation (*p* = 0.04) and indigestion syndrome (*p* = 0.02) No observed significant differences in the proportion of Roseburia and in abdominal pain (*p* = 0.13), reflux syndrome (*p* = 0.50) and diarrhoea (*p* > 0.05)	MedDiet adherence was assessed using the 14-item MEDAS questionnaire GI symptoms were assessed using the Gastrointestinal Symptom Rating Scale (GSRS) Medications for PD did not change during the study protocol Study funded as part of the University of Florida’s Creating the Healthiest Generation Moonshot initiative	Positive

**Table 3:** Study characteristics and summary of findings of the included cohort study.

Study (Country)	Study Design	Type of Intervention	Sample Size of Intervention Group	PD Definition	Mean Age, y	Males, %	PD Duration, y	Adjustment	Main Findings—MedDiet Associated with:	Additional Notes	Quality Assessment Rating
Fox et al., (2022) [46] (USA)	Cohort	Adherence to the MedDiet	1205	Not specified	66.4 ± 8.76	39	7.19 ± 5.44	Age, gender, income, and years since diagnosis	Decrease in PRO-PD score by 13.0 points (19.1–6.94) for non-motor symptoms and by 9.78 (14.3–5.23) for motor symptoms for each 1-point increase in the MEDAS score (*p* < 0.001)	MedDiet adherence was assessed using the MEDAS questionnaire. Study was independently funded.	Positive

**Table 4:** Risk of bias table for included studies with colour codes (Green indicates positive, orange indicates neutral and red indicates negative).

Relevance Questions	Study 1 (Paknahad et al., 2020 [43]) (Paknahad et al., 2022 [44])	Study 2 (Fox et al., 2022 [46])	Study 3 (Rusch et al., 2021 [45])
1. Would implementing the studied intervention or procedure (if found successful) result in improved outcomes for the patients/ clients/ population group?			
2. Did the authors study an outcome (dependent variable) or topic that the patients/ clients/ population would care about?			
3. Is the focus of the intervention or procedure (independent variable) or topic of study a common issue of concern to dietetics practice?			
4. Is the intervention or procedure feasible? (NA for some epidemiological studies)			
**Validity Questions**			
1. Was the research question clearly stated?			
2. Was the selecion of study subjects/ patients free from bias?			
3. Were study groups comparable?			
4. Was method of handling withdrawals described?			
5. Was blinding used to prevent introduction of bias?			
6. Were intervention/ therapeutic regimens/ exposure factor or procedure and any comparison(s) described in detail? Were intervening factors described?			
7. Were outcomes clearly defined and the measurements valid and reliable?			
8. Was the statistical analysis appropriate for the study design and type of outcome indicators?			
9. Are conclusions supported by results with biases and limitations taken into consideration?			
10. Is bias due to study’s funding or sponsorship unlikely?			
**Overall Rating**			

## Supplementary Materials

The following supporting information can be downloaded at: https://www.mdpi.com/article/10.3390/nu16142181/s1. Table S1: Search strategy for Ovid, EMBASE and EMCare. Table S2: Table of studies reviewed for inclusion. Table S3: Characteristics of excluded studies.
