# Supplementary material for: Mediterranean Diet Adherence, Gut Microbiota and Parkinson’s Disease: A Systematic Review
Source: Nutrients. 2024 Jul 9;16(14):2181. doi: 10.3390/nu16142181 (PMC11280252; doi:10.3390/nu16142181)
Supplement: Supplementary file 1 [file nutrients-16-02181-s001.zip › nutrients-3000796-supplementary.pdf]

Supplementary Material

Search strategy

Table S1. Search strategy for Ovid, EMBASE and EMCare

Search strategy	Number of publications retrieved		
	OVID MEDLINE	EMBASE	EMCare
1. Cretan* diet.mp.	11	15	7
2. Mediterranean* diet*.mp.	7898	14626	5333
3. Mediterranean* adj3 diet*).mp	9175	15075	5562
4. MedDiet*.mp	632	874	365
5. MD* diet*	119	169	55
6. (MEDI* adj3 diet*).mp.	19535	29766	9618
7. Diet, Mediterranean/	5280	12347	2867
8. 1 or 2 or 3 or 4 or 5 or 6 or 7	19622	29905	9656
9. Parkinson* disease.mp	135756	219957	36268
10. (Parkinson* adj1 disease*).mp.	135850	220092	36289
11. exp parkinsonian disorders/ or exp lewy body disease/ or exp parkinson disease/ or exp parkinson disease, secondary/	98002	218560	26593
12. 9 or 10 or 11	142018	243100	39672
13. 8 and 12	116	286	61

Search strategy for Scopus (n=198): ((TITLE-ABS-KEY (″Cretan diet″) OR TITLE-ABS- KEY (″Mediterranean adj3 diet″) OR TITLE-ABS-KEY (″MedDiet″) OR TITLE-ABS- KEY (″ MD″) OR TITLE-ABS-KEY (″MEDI adj3 diet″) AND ((TITLE-ABS-
KEY (″Parkinson disease″) OR TITLE-ABS-KEY (″Parkinson's disease"))

Search strategy for PubMed (n=76)

(((((((((((((("Mediterranean diet") AND (Parkinson disease)) OR (Mediterranean diet)) AND (Parkinson's disease)) OR (Mediterranean diet)) AND (PD)) OR (Mediterranean diet)) AND (Parkinsonism)) OR (MedDiet)) AND (Parkinson disease)) OR (MedDiet)) AND (Parkinson's disease)) OR (MedDiet)) AND (PD)) OR (MedDiet)) AND (Parkinsonism)

Inter-agreement for selected studies

First review of studies received an inter-agreement of 80%, with consensus reached in eight out of ten studies.

Table S2. Table of studies reviewed for inclusion. (Red colour indicates rejection)

Studies	First reviewer	Second reviewer
Fox et al., (2022)	Accept	Accept
Kyrozis et al., (2013)	Accept	Reject
Metcalfe-Roach et al., (2021)	Reject	Reject
Mischley et al., (2017)	Reject	Reject
Paknahad et al., (2020)	Accept	Accept
Paknahad et al., (2022)	Accept	Accept
Lawrie et al., (2022)	Accept	Reject
Alcalay et al., (2012)	Reject	Reject
Rusch et al., (2021)	Accept	Accept
Cassani et al., (2017)	Reject	Reject

Table S3. Characteristics of excluded studies

Studies	Reason for exclusion
Kyrozis et al., (2013)	Wrong outcome - Intervention did not include the key components of the MedDiet and focused on the effect of dairy intake on PD
Metcalfe-Roach et al., (2021)	Wrong outcome – study compared the effect of MIND diet vs.

	MedDiet in PD, rather than investigating the specific associations between the MedDiet and PD
Mischley et al., (2017)	Wrong outcome – Intervention assessed the individual effect of less than two MedDiet components on PD, and not MedDiet as a whole dietary pattern
Lawrie et al., (2022)	No MedDiet score available
Alcalay et al., (2012)	Wrong study design – retrospective study design
Cassani et al., (2017)	Less than two components of the MedDiet included
